# Supplementary material for: Information Overload, Wellbeing and COVID-19: A Survey in China
Source: Behav Sci (Basel). 2021 Apr 27;11(5):62. doi: 10.3390/bs11050062 (PMC8146850; doi:10.3390/bs11050062)
Supplement: Supplementary file 1 [file behavsci-11-00062-s001.zip › behavsci-1141062-supplementary.pdf]

Supplementary Material

1. **The Questionnaire**

COVID19 Wellbeing Questionnaire (English Version)

1. Signed Informed Consent form

2. Gender

Male
Female

3. Age

Under 18
18~25
26~30
31~40
41~50
51~60
Over 60

4. Current Location

_______(Province) ________(City)

5. Job

Student
Teacher
Medical and Nursing Staffs
Civil Servant
Office Worker
Factory Worker
Company Manger or Administer
Military Men or Policemen
Farmer
Freelancers
Self-employed
Retired
Unemployed
Other

6. Relationship status

Single
Married, none child
Married, one child or more
Divorced
Windowed

7. Role in COVID19 outbreak

Normal Public

Suspected Patient
Confirmed Patient
Recovered Patient
Close Contact with Confirmed case
Patient's family
Frontline Medical and Nursing Staffs
Other Frontline Workers (e.g., police officer, civil servant, volunteer)

8. Do you have family, friends, neighbours, or yourself were infected the COVID19?

I was infected

Family member was infected

Friend(s) or friend's family member was infected
Neighbours were infected (i.e., confirmed cases nearby the place you current live)
Myself, my family, friend or neighbours was suspected but not been infected
Myself, my family, friend or neighbours was close contact but not been infected
None

Coping type and personality (WPQ - Williams, Pendlebury, and Smith, 2017)

9. (Positive Coping) When I find myself in stressful situations I try to deal with it in a pro-active way (For example: by taking one step at a time, by changing something so that it would work out, by learning from the situation, by asking someone for help)

Disagree strongly 1 2 3 4 5 6 7 8 9 10 Agree strongly

10. (Negative Coping) When I find myself in stressful situations I tend to look inwardly (For example: I blame myself for the situation, wish that I had the power to change what has happened, wish the situation would go away, try to forget the whole thing)

Disagree strongly 1 2 3 4 5 6 7 8 9 10 Agree strongly

11. (Optimism) In general, I feel optimistic about the future (For example: I usually expect the best, I expect more good things to happen to me than bad, It's easy for me to relax)

Disagree strongly 1 2 3 4 5 6 7 8 9 10 Agree strongly

12. (Self-efficacy) I am confident in my ability to solve problems that I might face in life (For example: I can usually handle whatever comes my way, If I try hard enough I can overcome difficult problems, I can stick to my aims and accomplish my goals)

Disagree strongly 1 2 3 4 5 6 7 8 9 10 Agree strongly

13. (Self esteem) Overall, I feel that I have positive self-esteem (For example: On the whole I am satisfied with myself, I am able to do things as well as most other people, I feel that I am a person of worth)

Disagree strongly 1 2 3 4 5 6 7 8 9 10 Agree strongly

----

14. To what extent did you pay attention to the COVID19 epidemic?

Not at all 1 2 3 4 5 Very much

15. In the last two weeks, the average hour per day you spend on getting the COVID19 information, through the following options?

QQ (WhatsApp, Facebook, Messages, etc)
0 0.5 1 1.5 2 3 4 5 6 7 or more
WeChat (WhatsApp, Facebook, Messages, etc)
0 0.5 1 1.5 2 3 4 5 6 7 or more
Weibo (Twitter, etc)
0 0.5 1 1.5 2 3 4 5 6 7 or more
Other Social Media
0 0.5 1 1.5 2 3 4 5 6 7 or more
Video APPs (Tik Tok, Bilibili, Youtube, etc)
0 0.5 1 1.5 2 3 4 5 6 7 or more
Mobile News Apps
0 0.5 1 1.5 2 3 4 5 6 7 or more
Knowledge Sharing Website (Zhihu, Quora, etc)
0 0.5 1 1.5 2 3 4 5 6 7 or more
TV
0 0.5 1 1.5 2 3 4 5 6 7 or more
Broadcasting and newspaper
0 0.5 1 1.5 2 3 4 5 6 7 or more
Other
0 0.5 1 1.5 2 3 4 5 6 7 or more

16. Before COVID19 outbreaks, the average hour per day you spend on media (e.g., social media, media APPs, TV, broadcasting, newspaper) was?

Less than 30mins
Around 1 hour
Around 2 hours
Around 3 hours
Around 4 hours
Around 5 hours
Around 6 hours
Around 7 hours
Around 8 hours
Around 9 hours
Around 10 hours
More than 10 hours

17. Overall, to what extent you feel panic due to the COVID19 outbreaks?

Not at all 1 2 3 4 5 6 7 8 9 10 Very much so

Perceived Information Overload (Misra and Stokols, 2011)

18. In the last two weeks, how often have you felt overwhelmed with the COVID19 updates you received?

0 – Never
1 – Almost never

2 – Sometimes
3 – Fairly often
4 – Very often

19. In the last two weeks, how often have you forgotten to respond to important messages, text or email?

0 – Never

1 – Almost never

2 – Sometimes

3 – Fairly often

4 – Very often

20. In the last two weeks, how often have you felt pressured to respond to messages, text or email quickly? (e.g., report your current location and health status)

0 – Never

1 – Almost never

2 – Sometimes

3 – Fairly often

4 – Very often

21. In the last two weeks, how often have you received more information, updates, cases stories about COVID19 than you can handle?

0 – Never

1 – Almost never

2 – Sometimes

3 – Fairly often

4 – Very often

22. In the last two weeks, how often have you felt that you spend too much time on paying attention on COVID19?

0 – Never

1 – Almost never

2 – Sometimes

3 – Fairly often

4 – Very often

23. In the last two weeks, how often have you felt pressured to manage several information and communication inputs about COVID199 at the same time?

0 – Never

1 – Almost never

2 – Sometimes

3 – Fairly often

4 – Very often

24. In the last two weeks, how often have you felt that you received too many COVID19 updated (e.g., news, applications pop-up, event notifications, personal messages, and status updates) to deal with?

0 – Never

1 – Almost never

2 – Sometimes

3 – Fairly often

4 – Very often

25. In the last two weeks, how often have you felt that you have receive more instant messages about COVID19 that you can handle?

0 – Never

1 – Almost never

2 – Sometimes

3 – Fairly often

4 – Very often

26. In the last two weeks, how often have you felt that your focusing on COVID19 leave you too little for recreational activities?

0 – Never

1 – Almost never

2 – Sometimes

3 – Fairly often

4 – Very often

27. In the last two weeks, how often have you felt that your focusing on COVID19 information makes you less sensitive to the needs of others (e.g., eat or sleep)?

0 – Never

1 – Almost never

2 – Sometimes

3 – Fairly often

4 – Very often

28. In the last month, how often have you felt hassled by have to stay at home, or have to go out (e.g., for purchase or work)?

0 – Never

1 – Almost never

2 – Sometimes

3 – Fairly often

4 – Very often

29. In the last two weeks, how often have you felt that the extra demands on you due to COVID19 exceed your capacity to deal with them? (e.g., looking for masks and disinfection supplies, doing disinfection cleaning, extra work task, overtime work)

0 – Never

1 – Almost never

2 – Sometimes

3 – Fairly often

4 – Very often

30. In the last two weeks, how often have you felt that the environment surrounding you is too noisy?

0 – Never

1 – Almost never

2 – Sometimes

3 – Fairly often

4 – Very often

31. In the last two weeks, how often have you received the updated information about masks and disinfection supplies restocking?

0 – Never

1 – Almost never

2 – Sometimes

3 – Fairly often

4 – Very often

32. In the last two weeks, how often have you suffered setbacks on buying masks or disinfection supplies? (for example, out of stock, order cancelled, fake, etc.) *

0 – Never

1 – Almost never

2 – Sometimes

3 – Fairly often

4 – Very often

33. In the last two weeks, how often have you felt that you wanted to escape from network and media to reduce your attention on COVID19?

0 – Never

1 – Almost never

2 – Sometimes

3 – Fairly often

4 – Very often

Outcomes (WPQ - Williams, Pendlebury, and Smith, 2017)

34. (Positive affect) Facing COVID19 information and updated, in general, I mostly experience positive feelings (For example: I feel alert, inspired, determined, attentive)

Disagree strongly 1 2 3 4 5 6 7 8 9 10 Agree strongly

35. (Negative affect) Facing COVID19 information and updated, in general, I mostly experience negative feelings (For example: I feel upset, hostile, ashamed, nervous)

Disagree strongly 1 2 3 4 5 6 7 8 9 10 Agree strongly

36. (Life Satisfaction) Overall, I feel that I am satisfied with my life during COVID19 outbreaks (For example: In most ways my life is close to my ideal, so far I have gotten the important things I want)

Disagree strongly 1 2 3 4 5 6 7 8 9 10 Agree strongly

37. (Depression) On a scale of one to ten, how depressed would you say you are in general due to COVID19? (e.g. feeling 'down', no longer looking forward to things or enjoying things that you

used to)

Disagree strongly 1 2 3 4 5 6 7 8 9 10 Agree strongly

38. (Anxiety) On a scale of one to ten, how anxious would you say you are in general due to COVID19? (e.g. feeling tense or 'wound up', unable to relax, feelings of worry or panic)?

Disagree strongly 1 2 3 4 5 6 7 8 9 10 Agree strongly

39. (Stress) In general, how stressed did you find the COVID19 outbreak made you?

Disagree strongly 1 2 3 4 5 6 7 8 9 10 Agree strongly

40. Currently, how will you rate your overall mental health during the COVID19 outbreaks happened?

Not healthy 1 2 3 4 5 6 7 8 9 10 Extremely healthy

Location of respondents: City

	Frequency	Percent	Valid Percent	Cumulative Percent
Bangkok	1	.1	.1	31.4
Beijing	56	4.5	4.5	35.9
Bengbu	1	.1	.1	36.0
Changsha	9	.7	.7	36.7
Chongqing	11	.9	.9	37.6
Fuyang	3	.2	.2	37.8
Guangdong Cities	111	9.0	9.0	46.8
Guangzhou	72	5.8	5.8	52.6
Haerbing	2	.2	.2	52.7
Hangzhou	38	3.1	3.1	55.8
HangZhou	1	.1	.1	55.9
Hefei	7	.6	.6	56.5
Hubei Cities	90	7.3	7.3	63.7
Islamabad	1	.1	.1	63.8
Lower Hutt	2	.2	.2	64.0
Nanchang	1	.1	.1	64.0
Ningbo	8	.6	.6	65.0
Shanghai	61	4.9	4.9	70.0
Shenzhen	303	24.4	24.4	94.4
Wenzhou	13	1.0	1.0	95.9
Wuhan	38	3.1	3.1	99.0
Xi' an	1	.1	.1	99.0
Xinyang	1	.1	.1	99.1
Zhejiang cities	11	.9	.9	100.0
Total	1240	100.0	100.0	

Province

		Frequency	Percent	Valid Percent	Cumulative Percent
Valid	Anhui	19	1.5	1.5	1.5
	Beijing	56	4.5	4.5	6.0
	Chongqing	11	.9	.9	6.9
	Fujian	97	7.8	7.8	14.8
	Gansu	7	.6	.6	15.3
	Guangdong	486	39.2	39.2	54.5
	Guangxi	11	.9	.9	55.4
	Guizhou	2	.2	.2	55.6
	Hainan	6	.5	.5	56.0
	Hebei	12	1.0	1.0	57.0
	Heilongjiang	7	.6	.6	57.6
	Henan	20	1.6	1.6	59.2
	Hongkong	5	.4	.4	59.6
	Hubei	128	10.3	10.3	69.9
	Hunan	23	1.9	1.9	71.8
	Jiangsu	48	3.9	3.9	75.6
	Jiangxi	17	1.4	1.4	77.0
	Jilin	1	.1	.1	77.1
	Liaoning	9	.7	.7	77.8
	Neimenggu	5	.4	.4	78.2
	Ningxia	3	.2	.2	78.5
	Qinghai	1	.1	.1	82.7
	Shandong	24	1.9	1.9	84.7

	Shanghai	61	4.9	4.9	89.6
	Shanxi	15	1.2	1.2	90.8
	Shanxi2	15	1.2	1.2	92.0
	Sichuan	13	1.0	1.0	93.1
	Taiwan	1	.1	.1	93.1
	Tianjing	8	.6	.6	93.8
	Xinjiang	3	.2	.2	94.0
	Yunnan	3	.2	.2	94.3
	Zhejiang	71	5.7	5.7	100.0
	Total	1240	100.0	100.0	

Location Risk

		Frequency	Percent	Valid Percent	Cumulative Percent
Valid	Wuhan, Hubei	38	3.1	3.1	3.1
	Other cities in Hubei	90	7.3	7.3	10.3
	Cities with 300+ cases	516	41.6	41.6	51.9
	Cities with 100+ cases	72	5.8	5.8	57.7
	Cities near 300+ cities	122	9.8	9.8	67.6
	Cities near 100+ cities	63	5.1	5.1	72.7
	Other cities	287	23.1	23.1	95.8
	Total	1240	100.0	100.0	

Chinese version of the survey

新型冠状病毒肺炎疫情下心理负荷及应对措施调查

您好！近日，新型肺炎疫情牵动着全国人民的心，每天好坏消息层出不穷，给人们的心理造成了严重的影响。对此，深圳大学心理学院发起本次调查，旨在了解人们通过网络获取肺炎疫情相关信息时的心理和行为反应，以便为心理健康干预提供科学依据。本问卷采用**匿名调查**的形式，**严格遵守保密原则**，答案无对错之分，数据仅作科学研究之用。若您想知道调查的结果或愿意参与后续的追踪调研，您可选择在问卷最后留下您的联系方式。

本次问卷调查大约需时10分钟，您回答的真实性和完整性对我们后续心理干预策略的探究非常重要。非常感谢您的支持与参与！

本研究已通过深圳大学医学院伦理审查（ID: 2020007）如果您对此问卷有任何疑问，欢迎联系范老师（FanJL@szu.edu.cn）。

我已阅读并同意参与此研究 [单选题] *

○是，同意参与

○否，不同意参与

个人基本情况

您的性别： [单选题] *

○男 ○女

您的年龄段： [单选题] *

○18岁以下 ○18~25 ○26~30 ○31~40 ○41~50 ○51~60 ○60以上

您目前所在地： [填空题] *

____

您目前从事的职业： [单选题] *

○学生

○教师

○医护人员

○公务员

○普通职员（办公室、写字楼工作人员、白领）

○普通工人（工厂、体力劳动）

○企业管理人员（含基层、中高层）

○现役军人或警察

○农民（农林牧渔等）

○自由职业者

○个体经营者

○离退休人员

○无业人员

○其他

您的婚姻状况： [单选题] *

○未婚

○已婚无孩

○已婚有孩

○离异

○丧偶

您在此次疫情中的身份： [单选题] *

○普通民众

○疑似患者

○确诊患者

○治愈康复者

○密切接触者

○患者家属

○一线医护人员

○其他一线人员（如警察、公务员、志愿者等）

您的亲朋邻里是否有被感染新型冠状病毒？ [单选题] *

○自己被感染

○亲人被感染

○朋友或朋友亲属被感染

○邻居被感染（即您住宅附近有感染疑似或确诊病例）

○有疑似病例但未感染

○有密切接触但未被感染

○均未被感染

以下是对您个性的描述，0是该描述非常不符合您的实际情况，10是非常符合。

1. 总的来说，我对未来感到乐观。 [单选题] *

○完全不乐观	○1	○2	○3	○4	○5	○6	○7	○8	○9	○极其乐观

2. 我有信心可以解决生活中可能出现的问题。 [单选题] *

○完全没信心	○1	○2	○3	○4	○5	○6	○7	○8	○9	○极其有信心

3. 总的来说，我是有自尊有自信的人（如，对自己感到满意，觉得自己能跟多数人一样把事情办好，觉得自己很有价值）。 [单选题] *

○完全不是	○1	○2	○3	○4	○5	○6	○7	○8	○9	○极其如此

4. 我认为自己很外向（如，我很健谈，遇事可以泰然自若，对社交场合有自信）。 [单选题] *

○完全不外向	○1	○2	○3	○4	○5	○6	○7	○8	○9	○极其外向

5. 我觉得自己很有亲和力（如，对需要帮助的人有同情心，我喜欢对他人友好，善于合作）。 [单选题] *

○完全不亲和	○1	○2	○3	○4	○5	○6	○7	○8	○9	○极其亲和

6. 我觉得我是一个有责任心的人（如，准备充分，善于制定计划并执行，我注重细节）。 [单选题] *

○完全没有责任心	○1	○2	○3	○4	○5	○6	○7	○8	○9	○极其有责任心

7. 我觉得我可以跟别人相处融洽（我在他人面前很放松，从不嫉妒别人，我没有偏见、能完全接纳和尊重与自己截然不同的人——包括社会上的小众群体）。 [单选题] *

○完全不是	○1	○2	○3	○4	○5	○6	○7	○8	○9	○极其如此

第一部分

下面是关于您对新型冠状病毒肺炎疫情的***关注度***的一些题目，请您根据自身情况，如实作答。

您对当前肺炎疫情的关注度如何？ [单选题] *

○基本不关注

○偶尔关注

○一般

○比较关注

○非常关注

最近两周，您平均每天通过以下选项关注肺炎疫情的时间（小时）是？[矩阵量表题] *

	0	0.5	1	1.5	2	3	4	5	6	7或以上
社交平台										
QQ	○	○	○	○	○	○	○	○	○	○
微信	○	○	○	○	○	○	○	○	○	○
微博	○	○	○	○	○	○	○	○	○	○
其他社交平台	○	○	○	○	○	○	○	○	○	○
网络媒体										
视频软件（抖音、快手、Bilibili等）	○	○	○	○	○	○	○	○	○	○

手机新闻客户端（网易、今日头条等）	○	○	○	○	○	○	○	○	○	○
网络平台（知乎、豆瓣等）	○	○	○	○	○	○	○	○	○	○
传统媒体										
电视	○	○	○	○	○	○	○	○	○	○
广播和报纸	○	○	○	○	○	○	○	○	○	○
其他	○	○	○	○	○	○	○	○	○	○

肺炎疫情爆发之前，您平均每天使用媒体（手机、电视、广播）的时间是？ [单选题] *

○小于30分钟

○1小时左右

○2小时左右

○3小时左右

○4小时左右

○5小时左右

○6小时左右

○7小时左右

○8小时左右

○9小时左右

○10小时左右

○10小时以上

整体而言，肺炎疫情给您造成的恐慌或焦虑程度如何？ [单选题] *

○完全不恐慌、不焦虑	○1	○2	○3	○4	○5	○6	○7	○8	○9	○非常恐慌、焦虑

最近两周，您有多少次对您看到的疫情相关信息，感到不知所措？ [单选题] *

○完全没有 ○偶尔会 ○一般 ○经常会 ○一直如此

最近两周，您有多少次忘记回复重要的微信、短信或邮件？ [单选题] *

○完全没有 ○偶尔会 ○一般 ○经常会 ○一直如此

最近两周，您有多少次对必须回复微信、短信而感到有压力？（如，报备行踪和健康状况） [单选题] *

○完全没有 ○偶尔会 ○一般 ○经常会 ○一直如此

最近两周，您有多少次曾感到你了解到的疫情资讯、相关公告、分析性文章或社会动态，超出了您能理解或承受的范围？ [单选题] *

○完全没有 ○偶尔会 ○一般 ○经常会 ○一直如此

最近两周，您有多少次觉得自己在关注疫情资讯上花费了过多的时间？ [单选题] *

○完全没有 ○偶尔会 ○一般 ○经常会 ○一直如此

最近两周，您有多少次对不得不同时处理各种疫情资讯 和 即时通讯信息，而感到有压力？ [单选题] *

○完全没有 ○偶尔会 ○一般 ○经常会 ○一直如此

最近两周，您有多少次感到您接收了太多的疫情相关的推送（如新闻、APP推送、热搜、私人讯息、他人的朋友圈或QQ空间状态更新等）？ [单选题] *

○完全没有 ○偶尔会 ○一般 ○经常会 ○一直如此

最近两周，您有多少次感到您收到过多的关于疫情等即时通讯信息（如微信、QQ、群聊、短信等），超出了您能从容处理的范围？（如，不得不花费大量时间来理解、分析判断这些消息） [单选题] *

○完全没有 ○偶尔会 ○一般 ○经常会 ○一直如此

最近两周，您有多少次感到关注疫情导致您的娱乐活动时间被压缩减少？ [单选题] *

○完全没有 ○偶尔会 ○一般 ○经常会 ○一直如此

最近两周，您有多少次因为关注疫情，以至于你忽视了其他需求（如忽视饮食、睡觉的需求，废寝忘食等）？ [单选题] *

○完全没有 ○偶尔会 ○一般 ○经常会 ○一直如此

最近两周，您有多少次对不能出门、或不得不出门（如采购、办事或工作）而感到烦恼？ [单选题] *

○完全没有 ○偶尔会 ○一般 ○经常会 ○一直如此

最近两周，您有多少次感到因为疫情，有太多的事情需要做（如购买口罩、消毒打扫、或额外的工作），很难从容的处理？ [单选题] *

○完全没有　○偶尔会　○一般　○经常会　○一直如此

最近两周，您有多少次感到你身处的环境过于嘈杂？ [单选题] *

○完全没有　○偶尔会　○一般　○经常会　○一直如此

最近两周，您是否进行过防疫产品的采购（线上或线下，如口罩、消毒用品、药品等）？ [单选题] *

○完全没有　○偶尔会　○一般　○经常会　○一直如此

最近两周，您对防疫产品的消费是否遭到挫折（如无货、订单被取消、订购产品被征用、遇到无资质或不良商家等）？ [单选题] *

○完全没有　○偶尔会　○一般　○经常会　○一直如此

最近两周，您是否曾想过逃离网络和媒体，减少对疫情的关注？ [单选题] *

○完全没有　○偶尔会　○一般　○经常会　○一直如此

第二部分

下面的题目是关于新型冠状病毒肺炎**对您的影响**，请您根据自身情况如实作答。

当我感觉自己压力大的时候，我会尝试以积极主动的方式来应对（如，步步为营、从小事做起、从错误中学习、向他人求助） [单选题] *

○完全不是这样	○1	○2	○3	○4	○5	○6	○7	○8	○9	○完全如此

当我感觉自己压力大的时候，我会倾向于内省（如，责怪自己、希望我有能力改变事态、希望情况会自动消失、尝试忘记整件事情） [单选题] *

○完全不是这样	○1	○2	○3	○4	○5	○6	○7	○8	○9	○完全如此

面对疫情，我常有积极向上的感受（如，我感到被鼓舞，坚定，专注） [单选题] *

○完全不是这样	○1	○2	○3	○4	○5	○6	○7	○8	○9	○完全如此

面对疫情，我常有消极的感觉（如，我感到沮丧、不安、紧张、羞愧、对一些人或事态有敌意） [单选题] *

○完全不是这样	○1	○2	○3	○4	○5	○6	○7	○8	○9	○完全如此

总体而言，我对自己在疫情之下的生活感到满意（如，我的生活都接近理想的状况；我已经获得了生活中重要的东西） [单选题] *

○完全不是这样	○1	○2	○3	○4	○5	○6	○7	○8	○9	○完全如此

总体而言，因为疫情的动态，您会沮丧、不快乐吗？（如，情绪低落、不再期待或享受生活） [单选题] *

○完全不是这样	○1	○2	○3	○4	○5	○6	○7	○8	○9	○完全如此

总体而言，您觉得自己总体而言有多焦虑？（如，无法放松、紧张、恐慌、感到担心） [单选题] *

○完全不是这样	○1	○2	○3	○4	○5	○6	○7	○8	○9	○完全如此

总体而言，您觉得疫情带给你的压力有多大？ [单选题] *

○完全不是这样	○1	○2	○3	○4	○5	○6	○7	○8	○9	○完全如此

您认为当前肺炎疫情对您心理造成创伤程度是？ [单选题] *

○基本没有创伤

○轻度创伤

○中度创伤

○重度创伤

○极重度创伤

您认为2003年非典疫情对您心理造成的损伤程度是？ [单选题] *

○--我没有经历过SARS--

○基本没有创伤

○轻度创伤

○中度创伤

○重度创伤

○极重度创伤

您认为汶川大地震对您心理造成的损伤程度是？ [单选题] *

○--我没有经历过汶川大地震--

○基本没有创伤

○轻度创伤

○中度创伤

○重度创伤

○极重度创伤

当前，您认为自己在突发疫情之下，总体的心理健康程度如何？ [单选题] *

○并不健康	○1	○2	○3	○4	○5	○6	○7	○8	○9	○极其健康

在这次肺炎疫情期间，您是否有寻求过心理援助？ [单选题] *

○完全没有

○想过，但没有行动

○想过，但不知道相关途径

○有

感谢您的参与，您的作答对提高公民心理防护措施、促进健康心理建设有着重大意义。
若您想知道调查的结果、或愿意参与后续的追踪调研，您可在下面留下您的联系方式。我们将对您的联系方式进行加密处理，敬请放心。

您的手机号码： [填空题]

____

再次感谢您的参与。

如果您对此问卷有任何疑问，欢迎联系范老师（FanJL@szu.edu.cn）。

Factor analysis of COVID-Time items

Descriptive Statistics

	Mean	Std. Deviation[a]	Analysis N[a]	Missing N
Time_QQ	.474	1.1602	1240	0
Time_wechat	2.176	2.1301	1240	0
Time_weibo	1.450	2.0119	1240	0
Time_otherSNS	.933	1.6123	1240	0
Time_OnlineVideo	1.068	1.7238	1240	0
Time_OnlineNews	1.217	1.8416	1240	0
Time_website	.748	1.5044	1240	0
Time_TV	1.299	1.8473	1240	0
Time_BCnewspaper	.530	1.3860	1240	0
Time_Other	.483	1.1739	1240	0

Correlation Matrix

		Time_QQ	Time_wechat	Time_weibo	Time_otherSNS
Correlation	Time_QQ	1.000	.350	.318	.519
	Time_wechat	.350	1.000	.424	.485
	Time_weibo	.318	.424	1.000	.424
	Time_otherSNS	.519	.485	.424	1.000
	Time_OnlineVideo	.466	.458	.318	.552
	Time_OnlineNews	.479	.556	.263	.527
	Time_website	.547	.425	.414	.586
	Time_TV	.421	.547	.441	.518
	Time_BCnewspaper	.541	.359	.301	.471

	Time_Other	.502	.369	.227	.503
Sig. (1-tailed)	Time_QQ		.000	.000	.000
	Time_wechat	.000		.000	.000
	Time_weibo	.000	.000		.000
	Time_otherSNS	.000	.000	.000	
	Time_OnlineVideo	.000	.000	.000	.000
	Time_OnlineNews	.000	.000	.000	.000
	Time_website	.000	.000	.000	.000
	Time_TV	.000	.000	.000	.000
	Time_BCnewspaper	.000	.000	.000	.000
	Time_Other	.000	.000	.000	.000

Correlation Matrix

		Time_OnlineVideo	Time_OnlineNews	Time_website	Time_TV
Correlation	Time_QQ	.466	.479	.547	.421
	Time_wechat	.458	.556	.425	.547
	Time_weibo	.318	.263	.414	.441
	Time_otherSNS	.552	.527	.586	.518
	Time_OnlineVideo	1.000	.506	.548	.532
	Time_OnlineNews	.506	1.000	.543	.566
	Time_website	.548	.543	1.000	.543
	Time_TV	.532	.566	.543	1.000
	Time_BCnewspaper	.482	.567	.579	.609
	Time_Other	.464	.491	.487	.467
Sig. (1-tailed)	Time_QQ	.000	.000	.000	.000
	Time_wechat	.000	.000	.000	.000
	Time_weibo	.000	.000	.000	.000
	Time_otherSNS	.000	.000	.000	.000
	Time_OnlineVideo		.000	.000	.000
	Time_OnlineNews	.000		.000	.000
	Time_website	.000	.000		.000
	Time_TV	.000	.000	.000	
	Time_BCnewspaper	.000	.000	.000	.000
	Time_Other	.000	.000	.000	.000

Correlation Matrix

		Time_BCnewspaper	Time_Other
Correlation	Time_QQ	.541	.502
	Time_wechat	.359	.369

	Time_weibo	.301	.227
	Time_otherSNS	.471	.503
	Time_OnlineVideo	.482	.464
	Time_OnlineNews	.567	.491
	Time_website	.579	.487
	Time_TV	.609	.467
	Time_BCnewspaper	1.000	.625
	Time_Other	.625	1.000
Sig. (1-tailed)	Time_QQ	.000	.000
	Time_wechat	.000	.000
	Time_weibo	.000	.000
	Time_otherSNS	.000	.000
	Time_OnlineVideo	.000	.000
	Time_OnlineNews	.000	.000
	Time_website	.000	.000
	Time_TV	.000	.000
	Time_BCnewspaper		.000
	Time_Other	.000	

KMO and Bartlett's Test

Kaiser-Meyer-Olkin Measure of Sampling Adequacy.		.915
Bartlett's Test of Sphericity	Approx. Chi-Square	5917.686
	df	45
	Sig.	.000

Communalities

	Initial	Extraction
Time_QQ	1.000	.503
Time_wechat	1.000	.458
Time_weibo	1.000	.295
Time_otherSNS	1.000	.595
Time_OnlineVideo	1.000	.543
Time_OnlineNews	1.000	.585
Time_website	1.000	.619
Time_TV	1.000	.609
Time_BCnewspaper	1.000	.593
Time_Other	1.000	.506

Extraction Method: Principal Component Analysis.

Total Variance Explained

	Initial Eigenvalues			Extraction Sums of Squared Loadings	
Component	Total	% of Variance	Cumulative %	Total	% of Variance
1	5.306	53.064	53.064	5.306	53.064
2	.950	9.503	62.566		
3	.713	7.132	69.698		
4	.600	5.996	75.695		
5	.520	5.197	80.892		
6	.489	4.891	85.783		
7	.432	4.318	90.101		
8	.373	3.732	93.833		

9	.343	3.429	97.262		
10	.274	2.738	100.000		

Total Variance Explained

Component	Extraction Sums of Squared Loadings<br>Cumulative %
1	53.064
2	
3	
4	
5	
6	
7	
8	
9	
10	

Extraction Method: Principal Component Analysis.

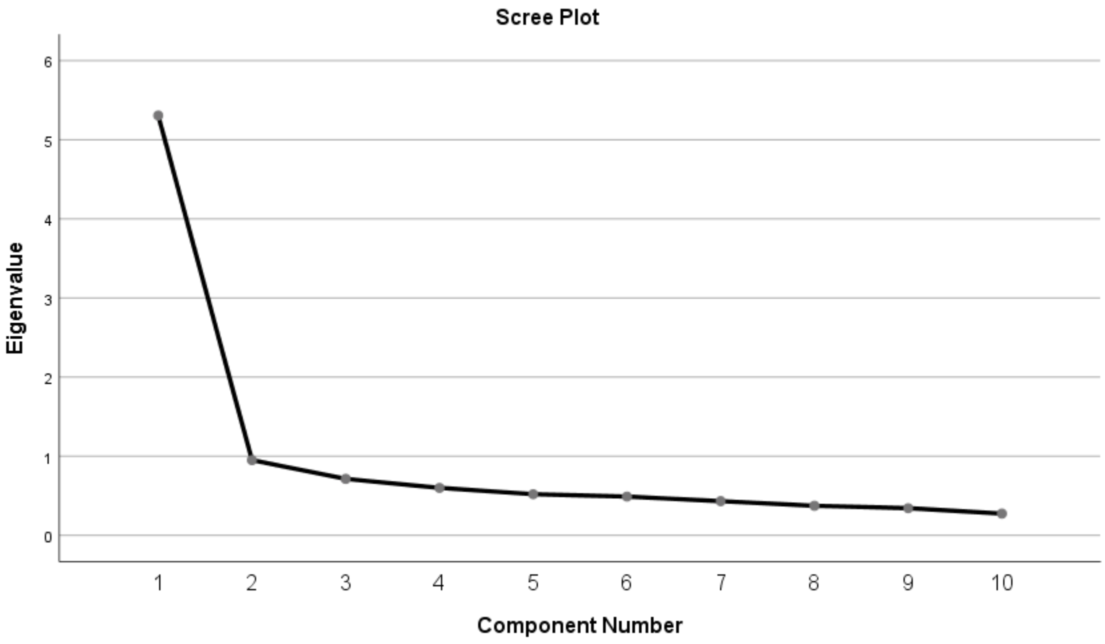

Component Matrix[a]

	Component
	1
Time_website	.787
Time_TV	.780
Time_otherSNS	.771
Time_BCnewspaper	.770
Time_OnlineNews	.765
Time_OnlineVideo	.737
Time_Other	.711
Time_QQ	.709
Time_wechat	.677
Time_weibo	.543

Extraction Method: Principal Component Analysis.[a]

a. 1 components extracted.

Component Score Coefficient Matrix

	Component
	1
Time_QQ	.134
Time_wechat	.128
Time_weibo	.102
Time_otherSNS	.145
Time_OnlineVideo	.139
Time_OnlineNews	.144
Time_website	.148
Time_TV	.147
Time_BCnewspaper	.145
Time_Other	.134

Descriptive Statistics

	Mean	Std. Deviation[a]	Analysis N[a]	Missing N
Q1_overwhelmed	2.53	1.190	1240	0
Q4_received_too_much_info	2.37	1.183	1240	0
Q5_spend_too_much_time	2.42	1.271	1240	0
Q6_pressured_to_manage_at_same_time	2.12	1.151	1240	0
Q16_escape	1.96	1.179	1240	0
Q12_additional_demands	2.45	1.200	1240	0
Q10_lower_sensitivity	1.91	1.087	1240	0
Q9_less_time_for_leisure	2.50	1.285	1240	0
Q8_instant_messages	2.37	1.198	1240	0
Q7_online_social_network	2.72	1.303	1240	0

Correlation Matrix

		Q1_overwhelmed	Q4_received_too_much_info	Q5_spend_too_much_time
Correlation	Q1_overwhelmed	1.000	.552	.423
	Q4_received_too_much_info	.552	1.000	.518
	Q5_spend_too_much_time	.423	.518	1.000
	Q6_pressured_to_manage_at_same_time	.477	.584	.624
	Q16_escape	.369	.426	.431
	Q12_additional_demands	.381	.432	.413
	Q10_lower_sensitivity	.387	.473	.504
	Q9_less_time_for_leisure	.311	.349	.416
	Q8_instant_messages	.425	.539	.575
	Q7_online_social_network	.330	.441	.544

Sig. (1-tailed)	Q1_overwhelmed		.000	.000
	Q4_received_too_much_info	.000		.000
	Q5_spend_too_much_time	.000	.000	
	Q6_pressured_to_manage_at_same_time	.000	.000	.000
	Q16_escape	.000	.000	.000
	Q12_additional_demands	.000	.000	.000
	Q10_lower_sensitivity	.000	.000	.000
	Q9_less_time_for_leisure	.000	.000	.000
	Q8_instant_messages	.000	.000	.000
	Q7_online_social_network	.000	.000	.000

Correlation Matrix

		Q6_pressured_to_manage_at_same_time	Q16_escape	Q12_additional_demands
Correlation	Q1_overwhelmed	.477	.369	.381
	Q4_received_too_much_info	.584	.426	.432
	Q5_spend_too_much_time	.624	.431	.413
	Q6_pressured_to_manage_at_same_time	1.000	.496	.484
	Q16_escape	.496	1.000	.400
	Q12_additional_demands	.484	.400	1.000
	Q10_lower_sensitivity	.568	.467	.512
	Q9_less_time_for_leisure	.426	.370	.466
	Q8_instant_messages	.653	.501	.472
	Q7_online_social_network	.553	.441	.383
Sig. (1-tailed)	Q1_overwhelmed	.000	.000	.000
	Q4_received_too_much_info	.000	.000	.000
	Q5_spend_too_much_time	.000	.000	.000
	Q6_pressured_to_manage_at_same_time		.000	.000
	Q16_escape	.000		.000
	Q12_additional_demands	.000	.000	
	Q10_lower_sensitivity	.000	.000	.000
	Q9_less_time_for_leisure	.000	.000	.000
	Q8_instant_messages	.000	.000	.000
	Q7_online_social_network	.000	.000	.000

Correlation Matrix

		Q10_lower_sensitivity	Q9_less_time_for_leisure	Q8_instant_messages

Correlation	Q1_overwhelmed	.387	.311	.425
	Q4_received_too_much_info	.473	.349	.539
	Q5_spend_too_much_time	.504	.416	.575
	Q6_pressured_to_manage_at_same_time	.568	.426	.653
	Q16_escape	.467	.370	.501
	Q12_additional_demands	.512	.466	.472
	Q10_lower_sensitivity	1.000	.525	.500
	Q9_less_time_for_leisure	.525	1.000	.497
	Q8_instant_messages	.500	.497	1.000
	Q7_online_social_network	.389	.444	.700
Sig. (1-tailed)	Q1_overwhelmed	.000	.000	.000
	Q4_received_too_much_info	.000	.000	.000
	Q5_spend_too_much_time	.000	.000	.000
	Q6_pressured_to_manage_at_same_time	.000	.000	.000
	Q16_escape	.000	.000	.000
	Q12_additional_demands	.000	.000	.000
	Q10_lower_sensitivity		.000	.000
	Q9_less_time_for_leisure	.000		.000
	Q8_instant_messages	.000	.000	
	Q7_online_social_network	.000	.000	.000

Correlation Matrix

		Q7_online_social_network
Correlation	Q1_overwhelmed	.330
	Q4_received_too_much_info	.441
	Q5_spend_too_much_time	.544

	Q6_pressured_to_manage_at_same_time	.553
	Q16_escape	.441
	Q12_additional_demands	.383
	Q10_lower_sensitivity	.389
	Q9_less_time_for_leisure	.444
	Q8_instant_messages	.700
	Q7_online_social_network	1.000
Sig. (1-tailed)	Q1_overwhelmed	.000
	Q4_received_too_much_info	.000
	Q5_spend_too_much_time	.000
	Q6_pressured_to_manage_at_same_time	.000
	Q16_escape	.000
	Q12_additional_demands	.000
	Q10_lower_sensitivity	.000
	Q9_less_time_for_leisure	.000
	Q8_instant_messages	.000
	Q7_online_social_network	

KMO and Bartlett's Test

Kaiser-Meyer-Olkin Measure of Sampling Adequacy.		.924
Bartlett's Test of Sphericity	Approx. Chi-Square	5793.996
	df	45
	Sig.	.000

Communalities

	Initial	Extraction
Q1_overwhelmed	1.000	.400
Q4_received_too_much_info	1.000	.541
Q5_spend_too_much_time	1.000	.576
Q6_pressured_to_manage_at_same_time	1.000	.673
Q16_escape	1.000	.451
Q12_additional_demands	1.000	.457
Q10_lower_sensitivity	1.000	.540
Q9_less_time_for_leisure	1.000	.430
Q8_instant_messages	1.000	.671
Q7_online_social_network	1.000	.529

Extraction Method: Principal Component Analysis.

Total Variance Explained

	Initial Eigenvalues			Extraction Sums of Squared Loadings	
Component	Total	% of Variance	Cumulative %	Total	% of Variance
1	5.268	52.678	52.678	5.268	52.678
2	.838	8.385	61.062		
3	.795	7.948	69.010		
4	.614	6.137	75.147		
5	.543	5.434	80.581		
6	.520	5.201	85.782		
7	.426	4.260	90.042		
8	.390	3.898	93.940		

9	.337	3.367	97.307		
10	.269	2.693	100.000		

Total Variance Explained

	Extraction Sums of Squared Loadings
Component	Cumulative %
1	52.678

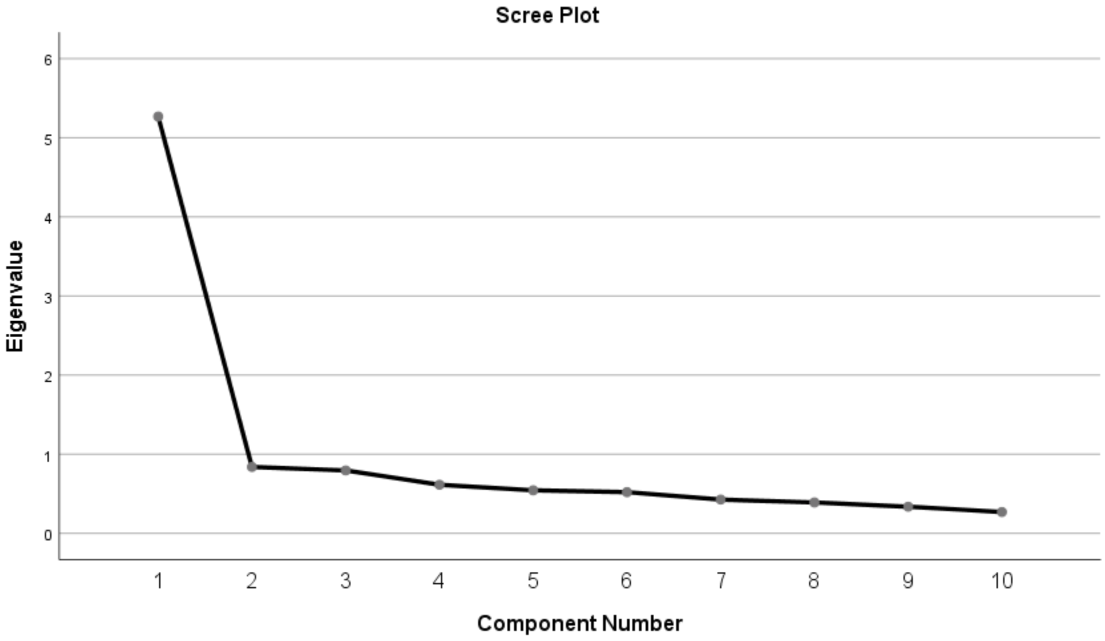

Component Matrix[a]

	Component
	1
Q6_pressured_to_manage_at_same_time	.820
Q8_instant_messages	.819
Q5_spend_too_much_time	.759

Q4_received_too_much_info	.735
Q10_lower_sensitivity	.735
Q7_online_social_network	.727
Q12_additional_demands	.676
Q16_escape	.672
Q9_less_time_for_leisure	.656
Q1_overwhelmed	.633

Extraction Method: Principal Component Analysis.[a]

a. 1 components extracted.

Component Score Coefficient Matrix

	Component
	1
Q1_overwhelmed	.120
Q4_received_too_much_info	.140
Q5_spend_too_much_time	.144
Q6_pressured_to_manage_at_same_time	.156
Q16_escape	.128
Q12_additional_demands	.128
Q10_lower_sensitivity	.140
Q9_less_time_for_leisure	.124
Q8_instant_messages	.155
Q7_online_social_network	.138

ANCOVAS EXAMINING EFFECTS OF COVID VARIABLES ON WELLBEING OUTCOMES

Between-Subjects Factors

		N
Percentile Group of COVID_IO	1	427
	2	411
	3	402

Descriptive Statistics

Dependent Variable: POSWB

Percentile Group of COVID_IO	Mean	Std. Deviation	N
1	22.0304	4.83983	427
2	19.5669	4.99973	411
3	18.1219	5.23155	402
Total	19.9468	5.27336	1240

Tests of Between-Subjects Effects

Dependent Variable: POSWB

Source	Type III Sum of Squares	df	Mean Square	F	Sig.	Partial Eta Squared
Corrected Model	15414.872[a]	10	1541.487	99.502	.000	.447
Intercept	875.510	1	875.510	56.514	.000	.044
NCOVID_I	276.933	2	138.467	8.938	.000	.014
GENIO	60.818	1	60.818	3.926	.048	.003
PSYCAP	4026.421	1	4026.421	259.904	.000	.175
NEGCOP	239.690	1	239.690	15.472	.000	.012

POSCOP	2259.021	1	2259.021	145.819	.000	.106
NPANIC_C	438.920	1	438.920	28.332	.000	.023
NCOVID_T	12.025	1	12.025	.776	.378	.001
NMASKS	.005	1	.005	.000	.985	.000
NCOVID_A	5.660	1	5.660	.365	.546	.000
Error	19039.615	1229	15.492			
Total	527818.000	1240				
Corrected Total	34454.487	1239				

a. R Squared = .447 (Adjusted R Squared = .443)

Estimated Marginal Means

Percentile Group of COVID_IO

Estimates

Dependent Variable: POSWB

Percentile Group of COVID_IO	Mean	Std. Error	95% Confidence Interval	
			Lower Bound	Upper Bound
1	20.805[a]	.233	20.348	21.263

2	19.648[a]	.195	19.266	20.031
3	19.340[a]	.252	18.845	19.835

a. Covariates appearing in the model are evaluated at the following values: GENIO = 8.1903, PSYCAP = 22.7258, NEGCOP = 6.28, POSCOP = 6.83, Percentile Group of PANIC_COVID = 1.93, Percentile Group of COVID_TIME = 2.00, Percentile Group of MASKS = 1.94, Percentile Group of COVID_ATT = 2.33.

Pairwise Comparisons

Dependent Variable: POSWB

(I) Percentile Group of COVID_IO	(J) Percentile Group of COVID_IO	Mean Difference (I-J)	Std. Error	Sig.[b]
1	2	1.157*	.298	.000
	3	1.465*	.401	.000
2	1	-1.157*	.298	.000
	3	.308	.326	.345
3	1	-1.465*	.401	.000
	2	-.308	.326	.345

Pairwise Comparisons

Dependent Variable: POSWB

(I) Percentile Group of COVID_IO	(J) Percentile Group of COVID_IO	95% Confidence Interval for Difference[b]	
		Lower Bound	Upper Bound
1	2	.573	1.741
	3	.679	2.251
2	1	-1.741	-.573
	3	-.332	.948
3	1	-2.251	-.679

2	-.948	.332

Based on estimated marginal means

*. The mean difference is significant at the .05 level.

b. Adjustment for multiple comparisons: Least Significant Difference (equivalent to no adjustments).

Univariate Tests

Dependent Variable: POSWB

	Sum of Squares	df	Mean Square	F	Sig.	Partial Eta Squared
Contrast	276.933	2	138.467	8.938	.000	.014
Error	19039.615	1229	15.492			

Between-Subjects Factors

		N
Percentile Group of COVID_IO	1	427
	2	411
	3	402

Descriptive Statistics

Dependent Variable: NEGWB

Percentile Group of COVID_IO	Mean	Std. Deviation	N
1	13.2014	6.71392	427
2	18.6399	7.36583	411
3	26.6940	6.97937	402

Total	19.3782	8.93938	1240

Tests of Between-Subjects Effects

Dependent Variable: NEGWB

Source	Type III Sum of Squares	df	Mean Square	F	Sig.	Partial Eta Squared
Corrected Model	53285.592[a]	10	5328.559	143.218	.000	.538
Intercept	2783.851	1	2783.851	74.823	.000	.057
NCOVID_I	4385.219	2	2192.609	58.932	.000	.088
GENIO	2193.799	1	2193.799	58.964	.000	.046
PSYCAP	2633.097	1	2633.097	70.771	.000	.054
NEGCOP	2475.668	1	2475.668	66.540	.000	.051
POSCOP	133.951	1	133.951	3.600	.058	.003
NPANIC_C	4078.152	1	4078.152	109.610	.000	.082
NCOVID_T	3.026	1	3.026	.081	.776	.000
NMASKS	1.183	1	1.183	.032	.859	.000
NCOVID_A	189.524	1	189.524	5.094	.024	.004
Error	45726.020	1229	37.206			
Total	564651.000	1240				
Corrected Total	99011.612	1239				

a. R Squared = .538 (Adjusted R Squared = .534)

Estimated Marginal Means

Percentile Group of COVID_IO

Estimates

Dependent Variable: NEGWB

Percentile Group of COVID_IO	Mean	Std. Error	95% Confidence Interval Lower Bound	Upper Bound
1	16.352[a]	.362	15.643	17.062
2	18.923[a]	.302	18.330	19.516
3	23.058[a]	.391	22.291	23.824

a. Covariates appearing in the model are evaluated at the following values: GENIO = 8.1903, PSYCAP = 22.7258, NEGCOP = 6.28, POSCOP = 6.83, Percentile Group of PANIC_COVID = 1.93, Percentile Group of COVID_TIME = 2.00, Percentile Group of MASKS = 1.94, Percentile Group of COVID_ATT = 2.33.

Pairwise Comparisons

Dependent Variable: NEGWB

(I) Percentile Group of COVID_IO	(J) Percentile Group of COVID_IO	Mean Difference (I-J)	Std. Error	Sig.[b]
1	2	-2.570*	.461	.000
	3	-6.705*	.621	.000
2	1	2.570*	.461	.000
	3	-4.135*	.505	.000

3	1	6.705*	.621	.000
	2	4.135*	.505	.000

Pairwise Comparisons

Dependent Variable: NEGWB

(I) Percentile Group of COVID_IO	(J) Percentile Group of COVID_IO	95% Confidence Interval for Difference[b] Lower Bound	Upper Bound
1	2	-3.475	-1.665
	3	-7.924	-5.487
2	1	1.665	3.475
	3	-5.127	-3.144
3	1	5.487	7.924
	2	3.144	5.127

Based on estimated marginal means

*. The mean difference is significant at the .05 level.

b. Adjustment for multiple comparisons: Least Significant Difference (equivalent to no adjustments).

Univariate Tests

Dependent Variable: NEGWB

	Sum of Squares	df	Mean Square	F	Sig.	Partial Eta Squared
Contrast	4385.219	2	2192.609	58.932	.000	.088
Error	45726.020	1229	37.206			

Between-Subjects Factors

	N

Percentile Group of COVID_ATT	1	124
	2	581
	3	535

Descriptive Statistics

Dependent Variable: NEGWB

Percentile Group of COVID_ATT	Mean	Std. Deviation	N
1	17.1532	7.67219	124
2	18.8571	8.57031	581
3	20.4598	9.45845	535
Total	19.3782	8.93938	1240

Tests of Between-Subjects Effects

Dependent Variable: NEGWB

Source	Type III Sum of Squares	df	Mean Square	F	Sig.	Partial Eta Squared
Corrected Model	53121.771[a]	10	5312.177	142.268	.000	.537
Intercept	1090.808	1	1090.808	29.213	.000	.023
NCOVID_A	200.899	2	100.450	2.690	.068	.004
GENIO	2308.260	1	2308.260	61.819	.000	.048
PSYCAP	2597.323	1	2597.323	69.560	.000	.054
NEGCOP	2517.609	1	2517.609	67.425	.000	.052
POSCOP	132.957	1	132.957	3.561	.059	.003
NPANIC_C	4169.623	1	4169.623	111.669	.000	.083
NCOVID_T	5.389	1	5.389	.144	.704	.000

NMASKS	2.063	1	2.063	.055	.814	.000
NCOVID_I	4210.037	1	4210.037	112.751	.000	.084
Error	45889.841	1229	37.339			
Total	564651.000	1240				
Corrected Total	99011.612	1239				

a. R Squared = .537 (Adjusted R Squared = .533)

Estimated Marginal Means

Percentile Group of COVID_ATT

Estimates

Dependent Variable: NEGWB

Percentile Group of COVID_ATT	Mean	Std. Error	95% Confidence Interval	
			Lower Bound	Upper Bound
1	18.588[a]	.566	17.477	19.699
2	19.128[a]	.255	18.628	19.628
3	19.833[a]	.271	19.302	20.364

a. Covariates appearing in the model are evaluated at the following values: GENIO = 8.1903, PSYCAP = 22.7258, NEGCOP = 6.28, POSCOP = 6.83, Percentile Group of PANIC_COVID = 1.93, Percentile Group of COVID_TIME = 2.00, Percentile Group of MASKS = 1.94, Percentile Group of COVID_IO = 1.98.

Pairwise Comparisons

Dependent Variable: NEGWB

(I) Percentile Group of COVID_ATT	(J) Percentile Group of COVID_ATT	Mean Difference (I-J)	Std. Error	Sig.[a]
1	2	-.540	.616	.381
	3	-1.245	.640	.052
2	1	.540	.616	.381
	3	-.705	.376	.061
3	1	1.245	.640	.052
	2	.705	.376	.061

Pairwise Comparisons

Dependent Variable: NEGWB

(I) Percentile Group of COVID_ATT	(J) Percentile Group of COVID_ATT	95% Confidence Interval for Difference[a]	
		Lower Bound	Upper Bound
1	2	-1.749	.668
	3	-2.501	.011
2	1	-.668	1.749
	3	-1.442	.033
3	1	-.011	2.501
	2	-.033	1.442

Based on estimated marginal means

a. Adjustment for multiple comparisons: Least Significant Difference (equivalent to no adjustments).

Univariate Tests

Dependent Variable: NEGWB

	Sum of Squares	df	Mean Square	F	Sig.	Partial Eta Squared
Contrast	200.899	2	100.450	2.690	.068	.004
Error	45889.841	1229	37.339			

Between-Subjects Factors

		N
Percentile Group of COVID_ATT	1	124
	2	581
	3	535

Descriptive Statistics

Dependent Variable: POSWB

Percentile Group of COVID_ATT	Mean	Std. Deviation	N
1	19.9113	5.18599	124
2	19.8744	5.02782	581
3	20.0336	5.55487	535
Total	19.9468	5.27336	1240

Tests of Between-Subjects Effects

Dependent Variable: POSWB

Source	Type III Sum of Squares	df	Mean Square	F	Sig.	Partial Eta Squared
Corrected Model	15377.473[a]	10	1537.747	99.066	.000	.446
Intercept	1505.342	1	1505.342	96.979	.000	.073
NCOVID_A	16.087	2	8.044	.518	.596	.001
GENIO	52.121	1	52.121	3.358	.067	.003
PSYCAP	4056.889	1	4056.889	261.357	.000	.175
NEGCOP	248.540	1	248.540	16.012	.000	.013
POSCOP	2263.302	1	2263.302	145.809	.000	.106
NPANIC_C	426.337	1	426.337	27.466	.000	.022
NCOVID_T	15.072	1	15.072	.971	.325	.001
NMASKS	.066	1	.066	.004	.948	.000
NCOVID_I	231.318	1	231.318	14.902	.000	.012
Error	19077.014	1229	15.522			
Total	527818.000	1240				
Corrected Total	34454.487	1239				

a. R Squared = .446 (Adjusted R Squared = .442)

Estimated Marginal Means

Percentile Group of COVID_ATT

Estimates

Dependent Variable: POSWB

Percentile Group of COVID_ATT	Mean	Std. Error	95% Confidence Interval	
			Lower Bound	Upper Bound
1	20.294[a]	.365	19.578	21.010
2	19.892[a]	.164	19.569	20.214
3	19.926[a]	.174	19.584	20.268

a. Covariates appearing in the model are evaluated at the following values: GENIO = 8.1903, PSYCAP = 22.7258, NEGCOP = 6.28, POSCOP = 6.83, Percentile Group of PANIC_COVID = 1.93, Percentile Group of COVID_TIME = 2.00, Percentile Group of MASKS = 1.94, Percentile Group of COVID_IO = 1.98.

Pairwise Comparisons

Dependent Variable: POSWB

(I) Percentile Group of COVID_ATT	(J) Percentile Group of COVID_ATT	Mean Difference (I-J)	Std. Error	Sig.[a]
1	2	.402	.397	.311
	3	.368	.413	.373
2	1	-.402	.397	.311
	3	-.034	.242	.887
3	1	-.368	.413	.373
	2	.034	.242	.887

Pairwise Comparisons

Dependent Variable: POSWB

(I) Percentile Group of COVID_ATT	(J) Percentile Group of COVID_ATT	95% Confidence Interval for Difference[a] Lower Bound	Upper Bound
1	2	-.377	1.182
	3	-.442	1.178
2	1	-1.182	.377
	3	-.510	.441
3	1	-1.178	.442
	2	-.441	.510

Based on estimated marginal means

a. Adjustment for multiple comparisons: Least Significant Difference (equivalent to no adjustments).

Univariate Tests

Dependent Variable: POSWB

	Sum of Squares	df	Mean Square	F	Sig.	Partial Eta Squared
Contrast	16.087	2	8.044	.518	.596	.001
Error	19077.014	1229	15.522			

Between-Subjects Factors

		N
Percentile Group of MASKS	1	444
	2	421
	3	375

Descriptive Statistics

Dependent Variable: POSWB

Percentile Group of MASKS	Mean	Std. Deviation	N
1	21.4955	5.16434	444
2	19.2280	4.89560	421
3	18.9200	5.39746	375
Total	19.9468	5.27336	1240

Tests of Between-Subjects Effects

Dependent Variable: POSWB

Source	Type III Sum of Squares	df	Mean Square	F	Sig.	Partial Eta Squared
Corrected Model	15444.300[a]	10	1544.430	99.847	.000	.448
Intercept	1443.037	1	1443.037	93.292	.000	.071
NMASKS	78.113	2	39.057	2.525	.080	.004
GENIO	51.036	1	51.036	3.299	.070	.003
PSYCAP	4041.489	1	4041.489	261.280	.000	.175
NEGCOP	237.325	1	237.325	15.343	.000	.012
POSCOP	2220.032	1	2220.032	143.524	.000	.105
NPANIC_C	431.143	1	431.143	27.873	.000	.022
NCOVID_T	13.942	1	13.942	.901	.343	.001
NCOVID_I	218.481	1	218.481	14.125	.000	.011
NCOVID_A	4.423	1	4.423	.286	.593	.000
Error	19010.188	1229	15.468			
Total	527818.000	1240				
Corrected Total	34454.487	1239				

a. R Squared = .448 (Adjusted R Squared = .444)

Estimated Marginal Means

Percentile Group of MASKS

Estimates

Dependent Variable: POSWB

Percentile Group of MASKS	Mean	Std. Error	95% Confidence Interval	
			Lower Bound	Upper Bound
1	20.118[a]	.208	19.710	20.527
2	19.596[a]	.192	19.218	19.973
3	20.138[a]	.225	19.697	20.579

a. Covariates appearing in the model are evaluated at the following values: GENIO = 8.1903, PSYCAP = 22.7258, NEGCOP = 6.28, POSCOP = 6.83, Percentile Group of PANIC_COVID = 1.93, Percentile Group of COVID_TIME = 2.00, Percentile Group of COVID_IO = 1.98, Percentile Group of COVID_ATT = 2.33.

Pairwise Comparisons

Dependent Variable: POSWB

(I) Percentile Group of MASKS	(J) Percentile Group of MASKS	Mean Difference (I-J)	Std. Error	Sig.[a]
1	2	.523	.288	.069
	3	-.020	.334	.953
2	1	-.523	.288	.069
	3	-.542	.292	.064
3	1	.020	.334	.953
	2	.542	.292	.064

Pairwise Comparisons

Dependent Variable: POSWB

(I) Percentile Group of MASKS	(J) Percentile Group of MASKS	95% Confidence Interval for Difference[a]	
		Lower Bound	Upper Bound
1	2	-.041	1.087
	3	-.675	.636
2	1	-1.087	.041
	3	-1.116	.031
3	1	-.636	.675
	2	-.031	1.116

Based on estimated marginal means

a. Adjustment for multiple comparisons: Least Significant Difference (equivalent to no adjustments).

Univariate Tests

Dependent Variable: POSWB

	Sum of Squares	df	Mean Square	F	Sig.	Partial Eta Squared

Contrast	78.113	2	39.057	2.525	.080	.004
Error	19010.188	1229	15.468			

Between-Subjects Factors

		N
Percentile Group of MASKS	1	444
	2	421
	3	375

Descriptive Statistics

Dependent Variable: NEGWB

Percentile Group of MASKS	Mean	Std. Deviation	N
1	15.2027	8.11288	444
2	19.8456	7.85205	421
3	23.7973	8.77566	375
Total	19.3782	8.93938	1240

Tests of Between-Subjects Effects

Dependent Variable: NEGWB

Source	Type III Sum of Squares	df	Mean Square	F	Sig.	Partial Eta Squared
Corrected Model	53123.401[a]	10	5312.340	142.278	.000	.537
Intercept	684.047	1	684.047	18.320	.000	.015
NMASKS	5.105	2	2.552	.068	.934	.000

GENIO	2309.009	1	2309.009	61.841	.000	.048
PSYCAP	2599.862	1	2599.862	69.631	.000	.054
NEGCOP	2507.716	1	2507.716	67.163	.000	.052
POSCOP	134.779	1	134.779	3.610	.058	.003
NPANIC_C	4166.091	1	4166.091	111.578	.000	.083
NCOVID_T	5.157	1	5.157	.138	.710	.000
NCOVID_I	4222.739	1	4222.739	113.095	.000	.084
NCOVID_A	199.797	1	199.797	5.351	.021	.004
Error	45888.211	1229	37.338			
Total	564651.000	1240				
Corrected Total	99011.612	1239				

a. R Squared = .537 (Adjusted R Squared = .533)

Estimated Marginal Means

Percentile Group of MASKS

Estimates

Dependent Variable: NEGWB

Percentile Group of MASKS	Mean	Std. Error	95% Confidence Interval	
			Lower Bound	Upper Bound
1	19.359[a]	.324	18.724	19.994
2	19.310[a]	.299	18.724	19.897
3	19.478[a]	.349	18.793	20.163

a. Covariates appearing in the model are evaluated at the following values: GENIO = 8.1903, PSYCAP = 22.7258, NEGCOP = 6.28, POSCOP = 6.83, Percentile Group of PANIC_COVID = 1.93, Percentile Group of COVID_TIME = 2.00, Percentile Group of COVID_IO = 1.98, Percentile Group of COVID_ATT = 2.33.

Pairwise Comparisons

Dependent Variable: NEGWB

(I) Percentile Group of MASKS	(J) Percentile Group of MASKS	Mean Difference (I-J)	Std. Error	Sig.[a]
1	2	.049	.447	.913
	3	-.119	.519	.819
2	1	-.049	.447	.913
	3	-.168	.454	.712
3	1	.119	.519	.819
	2	.168	.454	.712

Pairwise Comparisons

Dependent Variable: NEGWB

(I) Percentile Group of MASKS	(J) Percentile Group of MASKS	95% Confidence Interval for Difference[a]	
		Lower Bound	Upper Bound
1	2	-.828	.925
	3	-1.137	.899

2	1	-.925	.828
	3	-1.058	.723
3	1	-.899	1.137
	2	-.723	1.058

Based on estimated marginal means

a. Adjustment for multiple comparisons: Least Significant Difference (equivalent to no adjustments).

Univariate Tests

Dependent Variable: NEGWB

	Sum of Squares	df	Mean Square	F	Sig.	Partial Eta Squared
Contrast	5.105	2	2.552	.068	.934	.000
Error	45888.211	1229	37.338			

Between-Subjects Factors

		N
Percentile Group of COVID_TIME	1	413
	2	418
	3	409

Descriptive Statistics

Dependent Variable: NEGWB

Percentile Group of COVID_TIME	Mean	Std. Deviation	N
1	17.7070	8.32116	413

2	19.6890	8.56707	418
3	20.7482	9.64023	409
Total	19.3782	8.93938	1240

Tests of Between-Subjects Effects

Dependent Variable: NEGWB

Source	Type III Sum of Squares	df	Mean Square	F	Sig.	Partial Eta Squared
Corrected Model	53322.687[a]	10	5332.269	143.434	.000	.539
Intercept	679.486	1	679.486	18.278	.000	.015
NCOVID_T	207.692	2	103.846	2.793	.062	.005
GENIO	2391.010	1	2391.010	64.316	.000	.050
PSYCAP	2549.947	1	2549.947	68.592	.000	.053
NEGCOP	2523.983	1	2523.983	67.893	.000	.052
POSCOP	124.100	1	124.100	3.338	.068	.003
NPANIC_C	4235.840	1	4235.840	113.941	.000	.085
NCOVID_I	4207.262	1	4207.262	113.172	.000	.084
NCOVID_A	173.958	1	173.958	4.679	.031	.004
NMASKS	.506	1	.506	.014	.907	.000
Error	45688.925	1229	37.176			
Total	564651.000	1240				
Corrected Total	99011.612	1239				

a. R Squared = .539 (Adjusted R Squared = .535)

Estimated Marginal Means

Percentile Group of COVID_TIME

Estimates

Dependent Variable: NEGWB

Percentile Group of COVID_TIME	Mean	Std. Error	95% Confidence Interval Lower Bound	95% Confidence Interval Upper Bound
1	19.005[a]	.310	18.397	19.614
2	19.949[a]	.300	19.361	20.537
3	19.171[a]	.312	18.560	19.783

a. Covariates appearing in the model are evaluated at the following values: GENIO = 8.1903, PSYCAP = 22.7258, NEGCOP = 6.28, POSCOP = 6.83, Percentile Group of PANIC_COVID = 1.93, Percentile Group of COVID_IO = 1.98, Percentile Group of COVID_ATT = 2.33, Percentile Group of MASKS = 1.94.

Pairwise Comparisons

Dependent Variable: NEGWB

(I) Percentile Group of COVID_TIME	(J) Percentile Group of COVID_TIME	Mean Difference (I-J)	Std. Error	Sig.[b]
1	2	-.944*	.432	.029
	3	-.166	.452	.714

2	1	.944*	.432	.029
	3	.778	.433	.073
3	1	.166	.452	.714
	2	-.778	.433	.073

Pairwise Comparisons

Dependent Variable: NEGWB

(I) Percentile Group of COVID_TIME	(J) Percentile Group of COVID_TIME	95% Confidence Interval for Difference[b]	
		Lower Bound	Upper Bound
1	2	-1.791	-.096
	3	-1.053	.722
2	1	.096	1.791
	3	-.072	1.628
3	1	-.722	1.053
	2	-1.628	.072

Based on estimated marginal means

*. The mean difference is significant at the .05 level.

b. Adjustment for multiple comparisons: Least Significant Difference (equivalent to no adjustments).

Univariate Tests

Dependent Variable: NEGWB

	Sum of Squares	df	Mean Square	F	Sig.	Partial Eta Squared
Contrast	207.692	2	103.846	2.793	.062	.005
Error	45688.925	1229	37.176			

Between-Subjects Factors

		N
Percentile Group of COVID_TIME	1	413
	2	418
	3	409

Descriptive Statistics

Dependent Variable: POSWB

Percentile Group of COVID_TIME	Mean	Std. Deviation	N
1	20.1332	5.11510	413
2	19.5861	5.24219	418
3	20.1271	5.45332	409
Total	19.9468	5.27336	1240

Tests of Between-Subjects Effects

Dependent Variable: POSWB

Source	Type III Sum of Squares	df	Mean Square	F	Sig.	Partial Eta Squared
Corrected Model	15416.946[a]	10	1541.695	99.527	.000	.447
Intercept	1561.856	1	1561.856	100.828	.000	.076
NCOVID_T	64.899	2	32.449	2.095	.124	.003
GENIO	59.782	1	59.782	3.859	.050	.003
PSYCAP	4021.953	1	4021.953	259.644	.000	.174
NEGCOP	245.105	1	245.105	15.823	.000	.013
POSCOP	2240.004	1	2240.004	144.607	.000	.105

NPANIC_C	436.336	1	436.336	28.168	.000	.022
NCOVID_I	226.807	1	226.807	14.642	.000	.012
NCOVID_A	3.019	1	3.019	.195	.659	.000
NMASKS	.168	1	.168	.011	.917	.000
Error	19037.541	1229	15.490			
Total	527818.000	1240				
Corrected Total	34454.487	1239				

a. R Squared = .447 (Adjusted R Squared = .443)

Estimated Marginal Means

Percentile Group of COVID_TIME

Estimates

Dependent Variable: POSWB

Percentile Group of COVID_TIME	Mean	Std. Error	95% Confidence Interval	
			Lower Bound	Upper Bound
1	19.952[a]	.200	19.559	20.345
2	19.662[a]	.193	19.282	20.041

3	20.233[a]	.201	19.838	20.628

a. Covariates appearing in the model are evaluated at the following values: GENIO = 8.1903, PSYCAP = 22.7258, NEGCOP = 6.28, POSCOP = 6.83, Percentile Group of PANIC_COVID = 1.93, Percentile Group of COVID_IO = 1.98, Percentile Group of COVID_ATT = 2.33, Percentile Group of MASKS = 1.94.

Pairwise Comparisons

Dependent Variable: POSWB

(I) Percentile Group of COVID_TIME	(J) Percentile Group of COVID_TIME	Mean Difference (I-J)	Std. Error	Sig.[b]
1	2	.291	.279	.298
	3	-.281	.292	.337
2	1	-.291	.279	.298
	3	-.571*	.280	.041
3	1	.281	.292	.337
	2	.571*	.280	.041

Pairwise Comparisons

Dependent Variable: POSWB

(I) Percentile Group of COVID_TIME	(J) Percentile Group of COVID_TIME	95% Confidence Interval for Difference[b]	
		Lower Bound	Upper Bound
1	2	-.257	.838
	3	-.854	.292
2	1	-.838	.257
	3	-1.120	-.023
3	1	-.292	.854
	2	.023	1.120

Based on estimated marginal means

*. The mean difference is significant at the .05 level.

b. Adjustment for multiple comparisons: Least Significant Difference (equivalent to no adjustments).

Univariate Tests

Dependent Variable: POSWB

	Sum of Squares	df	Mean Square	F	Sig.	Partial Eta Squared
Contrast	64.899	2	32.449	2.095	.124	.003
Error	19037.541	1229	15.490			
